# Utilization of Clustered Regularly Interspaced Short Palindromic Repeats to Genotype *Escherichia coli* Serogroup O80

**DOI:** 10.3389/fmicb.2020.01708

**Published:** 2020-07-23

**Authors:** Jinzhao Long, Yake Xu, Liuyang Ou, Haiyan Yang, Yuanlin Xi, Shuaiyin Chen, Guangcai Duan

**Affiliations:** ^1^College of Public Health, Zhengzhou University, Zhengzhou, China; ^2^Institute for AIDS/STD Control and Prevention, Henan Province Center for Disease Control and Prevention, Henan, China; ^3^Henan Innovation Center of Molecular Diagnosis and Laboratory Medicine, Xinxiang Medical University, Xinxiang, China

**Keywords:** clustered regularly interspaced short palindromic repeat typing, Shiga toxin-producing *Escherichia coli* serogroup O80, multi-locus sequence typing, serotyping, virulence gene profiles

## Abstract

The hypervariable nature of clustered regularly interspaced short palindromic repeats (CRISPRs) makes them valuable biomarkers for subtyping and epidemiological investigation of *Escherichia coli*. Shiga toxin-producing *E. coli* (STEC) serogroup O80 is one hybrid pathotype that is emerging recently in Europe and is involved in hemolytic uremic syndrome with bacteremia. However, whether STEC O80 strains can be genotyped using CRISPR has not been evaluated. In this study, we aimed to characterize the genetic diversity of 81 *E. coli* serogroup O80 isolates deposited in the National Center for Biotechnology Information databases using CRISPR typing and to explore the association between virulence potential and CRISPR types (CTs). A total of 21 CTs were identified in 80 O80 strains. CRISRP typing provided discrimination with variants of a single serotype, which suggested a stronger discriminatory power. Based on CRISPR spacer profiles, 70 O80:H2 isolates were further divided into four lineages (lineage LI, LII, LIII, and LIV), which correlated well with whole-genome single nucleotide polymorphisms typing and virulence gene profiles. Moreover, the association between CRISPR lineages and virulence gene profiles hinted that STEC O80:H2 strains may originate from O80:H19 or O80:H26 and that lineage LI may have been evolved from lineage LII. CT2 and CT13 were shared by human and cattle isolates, suggesting that there might be the potential transmission between cattle and human. Collectively, CRISPR typing is one technology that can be used to monitor the transmission of STEC O80 strains and provide new insights into microevolution of serogroup O80.

## Introduction

Shiga toxin-producing *Escherichia coli* (STEC) is the major food-borne zoonotic pathogen related to both outbreaks and sporadic cases, representing a worldwide public health concern (Karmali, 2017). STEC infection in humans can cause a series of gastrointestinal diseases ranging from mild to severe diarrhea. One subset of the STEC strains, called as enterohemorrhagic *E. coli*, is specifically linked to life-threatening hemolytic uremic syndrome (HUS). HUS is characterized by acute kidney failure, thrombocytopenia, and hemolytic anemia (Karpman et al., 2017). To date, over 400 STEC serotypes have been detected in humans and animals, but only a small subset of serotypes is responsible for HUS cases (Delannoy et al., 2013). Historically, the serogroup O157 contributes to most HUS cases of STEC infection, but other non-O157 serogroups are increasingly important in public health (Espie et al., 2008).

The serogroup O80, especially serotype O80:H2, has been reported to be significantly linked to HUS with bacteremia, which has unusual features of STEC infection (Mariani-Kurkdjian et al., 2014). Indeed, it is a hybrid pathotype that combines many intestinal virulence factors (VFs; *stx*, *eae*, and *ehxA*) and extraintestinal virulence determinants (Cointe et al., 2018). The extraintestinal virulence phenotype is attributed to the presence of a mosaic plasmid that harbors multiple extraintestinal VFs (*sitABCD*, *iss*, *iutA*, *iucC*, *shiF*, *iroN*, *hlyF*, *ompTp*, and *mig-14*) and is closely related to the pathogenic plasmid pS88. The mosaic plasmid also encodes a multidrug resistance gene cassette, which confers resistance to multiple classes of antibiotics (e.g., aminopenicillin, aminoglycoside, tetracycline, and β-lactam). Recent data suggest that the serogroup O80 has become the second most frequent STEC serogroup isolated in 2016 in France (Bruyand et al., 2019). Moreover, this serogroup has also been isolated from cattle in Spain (Blanco et al., 2004), human and cattle in Belgium (De Rauw et al., 2019), and human in Switzerland (Fierz et al., 2017; Nuesch-Inderbinen et al., 2018), and Netherlands (Wijnsma et al., 2017). The prevalence and severity of this serogroup make it essential to establish effective typing technology used for monitoring the transmission of STEC O80 strains throughout the food chain and environment.

So far, several molecular subtyping technologies have been exploited to perform epidemiological surveillance of pathogenic *E. coli*, such as serotyping, multi-locus sequence typing (MLST), pulsed field gel electrophoresis (PFGE), and whole-genome sequence typing (WGST), but they all have some limitations. The serotyping method is confined to laboratory diagnosis because it needs skillful manipulation, intensive labor, and high cost. In particular, it cannot deduce phylogenetic relationship between strains (Orskov et al., 1977). Three MLST typing schemes for *E. coli* have been established based on three different sets of house-keeping genes (Reid et al., 2000; Wirth et al., 2006; Jaureguy et al., 2008). The matched online databases for the three typing schemes are hosted at the University of Warwick^1^, Institut Pasteur^2^, and Michigan State University^3^, which facilitate the exchange and comparison of typing results between different labs. The reproducibility and portability of MLST typing make it suitable for long-term epidemiology, but its low resolution hinders the correct identification of contamination source in short-term outbreak (Perez-Losada et al., 2013). PFGE offers an affordable alternative to MLST in outbreak investigations and exhibits a higher discriminatory power. However, the gel-based approach often requires a high level of expertise to interpret and translate binding patterns. WGST is increasingly used for pathogen diagnosis and outbreak tracing in advanced laboratories; nevertheless, high cost limits its widespread application in low-income countries.

Recently, there is another new methodology used for strain typing that is based on the characterization of clustered regularly interspaced short palindromic repeat (CRISPR) array (Barrangou and Dudley, 2016). It has been demonstrated that the CRISPR array together with CRISPR-associated proteins constitutes a prokaryotic adaptation immunity system against the invasion by mobile genetic elements (e.g., phages and plasmids; Barrangou et al., 2007). The CRISPR array is characterized by alternating repeat sequences separated by variable spacers of regular length. Theoretically, these hypervariable spacers are the remnants of foreign DNA invasion, which are incorporated into the CRISPR array in an ordinal manner (Bolotin et al., 2005). Thus, the combinations of these spacers result in strain-to-strain difference, which makes CRISPR loci applicable for pathogen identification and subtyping. To date, four CRISPR loci have been found in *E. coli*, CRISPR1 and CRISPR2 for type I–E CRISPR/Cas system and CRISPR3 and CRISPR4 for type I–F system (Diez-Villasenor et al., 2010). One research group has developed a CRISPR-targeting real-time polymerase chain reaction protocol to detect STEC strains of eight specific serotypes based on the association between CRISPR composition and serotype (Delannoy et al., 2012a, b). Also, another investigation found that the total number of spacers in CRISPR arrays negatively correlated with virulence potential of STEC strains (Toro et al., 2014). Thus, we hypothesize that the CRISPR arrays might be a suitable biomarker to genotype O80 strains, and there might be a correlation between CRISPR types (CTs) and virulence gene profiles.

To test hypothesis, we characterized *in silico* the genetic diversity of 81 serogroup O80 strains using CRISPR typing, investigated the relationships between CRISPR typing and other typing methods, including serotyping, MLST, and whole-genome single nucleotide polymorphism (wgSNP) typing, and explored the potential association between CTs and virulence gene profiles.

## Materials and Methods

### Bacterial Isolates and Metadata

*Escherichia coli* O80 strains previously sequenced and deposited in the National Center for Biotechnology Information (NCBI) databases were used in this study (*n* = 81; retrieved in July 2019). All the information associated with O80 strains (e.g., geographical origin, host source, and year of isolation) were collected either from published papers or NCBI databases. Also, 12 reference STEC strains available in NCBI databases were also included in this study for comparative analysis. Detailed information on metadata, WGS accessions, sequencing coverage, and assembly N50 values and references for all strains used in this study were provided in Supplementary Table S1.

### Sequence Assembly and Annotation

The sequences of 81 O80 strains were deposited in NCBI^4^. Of them, 65 strains contained assembled genome sequences (complete genomes or draft genomes), and 16 strains only contained raw sequencing reads from Sequence Read Archive. Assembled genome sequences and raw sequencing data were downloaded from the NCBI databases. For those strains that contain unassembled genomes, the Unicycler version 0.4.3 open-source software was used to assemble raw sequencing reads of each strain into a single “fasta” file for subsequent analysis (Wick et al., 2017). The coverage of all genomes was more than 20-fold. All genomes were annotated using RAST web server^5^ (Aziz et al., 2008).

### Multi-Locus Sequence Typing, Serotyping, Two-Locus Clonal Typing, and Whole-Genome Single Nucleotide Polymorphisms Analysis

*In silico* analysis of MLST, serotyping, and two-locus clonal typing were performed by MLST 2.0, SerotypeFinder 2.0, and CHTyper 1.0 available on the CGE website, respectively^6^. The MLST typing scheme utilized the seven house-keeping genes (*adk*, *icd*, *fumC*, *purA*, *gyrB*, *mdh*, and *recA*), and the sequences of seven genes were concatenated to construct a neighbor-joining tree (Wirth et al., 2006). The combination of two genes (*fumC* and *fimH*) was used for two-locus clonal typing (Weissman et al., 2012). The wgSNP analysis was performed by CSI Phylogeny 1.4 on the CGE website^7^ (Kaas et al., 2014). The phylogenetic trees based on *cas1* gene, MLST, and wgSNPs were generated by MEGA version 7.0 using the neighbor-joining or maximum likelihood method.

### Clustered Regularly Interspaced Short Palindromic Repeat Identification, Typing, Visualization, and Clustering

All the genomes were submitted to CRISPR Recognition Tool to identify CRISPR arrays and extract spacer sequences (Bland et al., 2007). The settings were as follows: repeat length 28–30 nt, spacer length 29–34 nt, minimum repeats per array 3, and search window 8. Such strict parameters could filter out “questionable” CRISPR but often miss the last repeat/spacer combination as the last repeat was degenerated. The last spacer/repeat combination was blasted against corresponding genome, and then, it was manually added once confirmed. Meanwhile, irregular repeat sequences were manually modified to ensure the comparability of spacers if necessary. For genomes with a truncated CRISPR array, CRISPR sequence was re-extracted by BLAST using a conserved repeat sequence against whole-genome shotgun contigs. Truncated CRISPR arrays were reassembled and aligned using SeqMan (Lasergene 7.1; DNAStar) to recover the complete CRISPR loci. One truncated and unrecoverable CRISPR was removed for subsequent analysis.

Subsequently, each spacer was blasted against the spacer dictionary constructed previously by Yin et al. (2013) to obtain the name of the spacers (covered length = 100% and identity = 100%). According to the numbering system described previously by Yin et al. (2013), a new number was assigned for spacers that had no perfect match in the spacer dictionary, and then, a CRISPR allele was defined by each unique spacer combination within a CRISPR locus. For alleles not previously introduced by Yin et al. (2013), a new CRISPR allele number was designated. In parallel, a CT was assigned based on each unique CRISPR1 and CRISPR2 allele combination. The visualization of CRISPR arrays was accomplished by the CRISPRstudio tool (Dion et al., 2018). The presence or absence of every spacer in CRISPR arrays for each strain was used to create a binary library. Simply, if a spacer was present in a strain, it was designated as “1”; otherwise, it was designated as “0.” The binary patterns of all isolates were uploaded to Phyloviz version 2.0 to establish a minimum spanning tree generated by the goeBURST algorithm (Nascimento et al., 2017).

### *In silico* Determination of Virulence Gene Profiles and Plasmid Content

*In silico* determination of virulence genes was performed by VFanalyzer on the VFs Database^8^. Additional VFs (i.e., *sitA*, *sitB*, *sitC*, *sitD*, *iss*, *iutA*, *iucC*, *shiF*, *iroN*, *hlyF*, *ompTp*, and *mig-14*) associated with the extraintestinal virulence plasmid pS88 were searched by BLAST + version 2.9.0 (Camacho et al., 2009). The presence of plasmid pRDEx444_B (accession number: QBDM01000003.1) was also determined by the BLAST tool (coverage ≧ 85%, identity ≧ 95%).

### Statistical Analysis

Results was analyzed with SPSS 21.0. Chi square and Fisher’s exact test were used for the comparison of the distribution differences of *stx2d* gene and HUS cases among four CRISPR lineages. In all cases, a *p*-value lower than 0.05 was deemed as significant.

## Results

### Serotyping, Two-Locus Clonal Typing, and Multi-Locus Sequence Typing of *Escherichia coli* O80 Strains

Based on the results of SerotypeFinder, 81 O80 strains examined were positive for the serogroup O80 *wzx* (*wzx*_O80_) or *wzy* (*wzy*_O80_) gene. Of these, 70 strains were positive for the H2 *fliC* (*fliC*_H2_) gene, 8 strains were positive for the H26 *fliC* (*fliC*_H26_) gene, and 3 strains were positive for the H19 *fliC* (*fliC*_H19_) gene. Thus, they were O80:H2, O80:H26, and O80:H19 strains, respectively. Additionally, all the O80:H2 strains, 7 O80:H26 strains, and 1 O80:H19 strain were clonotype (CH) 27–54 based on wo-locus (*fumC*/*fimH*) clonal typing. The remaining 1 O80:H26 strain was CH 27–30 and 2 O80:H19 strains were CH 27–23 (Supplementary Table S1).

**FIGURE 1:**
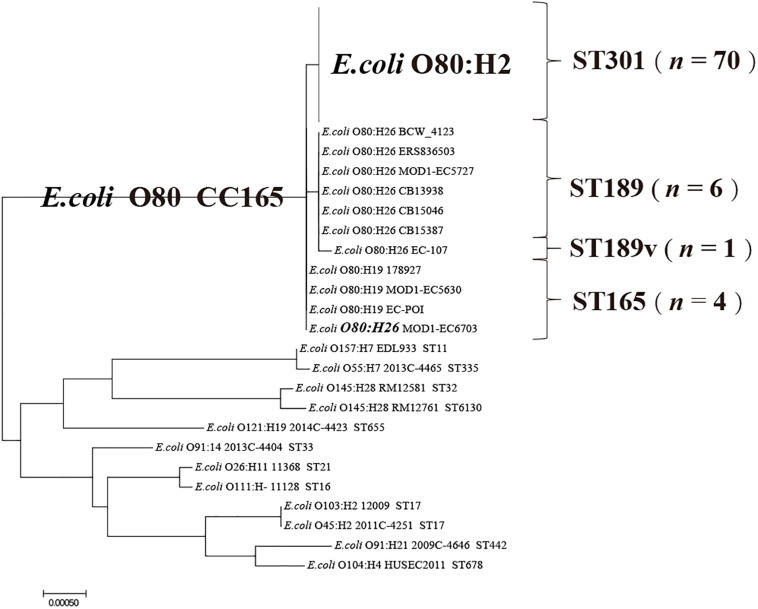
Phylogenetic analysis of STEC O80 strains based on MLST as well as 12 reference STEC strains. The sequences of seven house-keeping genes (*adk, icd, fumC, purA, gyrB, icd*, and *recA*) from STEC O80 strains and 12 reference STEC strains are concentrated to construct a neighbor-joining tree.

Multi-locus sequence typing results showed that all the O80:H2 strains were sequence type (ST) 301, all the O80:H19 strains and 1 O80:H26 strain were ST165, and 6 O80:H26 strains were ST189 (Figure 1 and Supplementary Table S1). Strain EC-107 could not be assigned an ST because there was no perfect match to the existing *gyrB* allele. Phylogenetic analysis based on MLST revealed that this strain was the variant of ST189 that contained a non-synonymous A → C transition in *gyrB*. As shown in Figure 1, all the O80 strains formed a single clonal complex (CC165).

### Clustered Regularly Interspaced Short Palindromic Repeat Typing of *Escherichia coli* O80 Strains

All the O80 strains were analyzed for the occurrence and diversity of CRISPR/Cas system. One typical type I–E CRISPR/Cas system, including CRISPR1, CRISPR2a arrays, and a set of *cas* genes (*cas3-cse1-cse2-cas7-cas5-cas6e-cas1-cas2*), was found in all the O80 strains.

**FIGURE 2:**
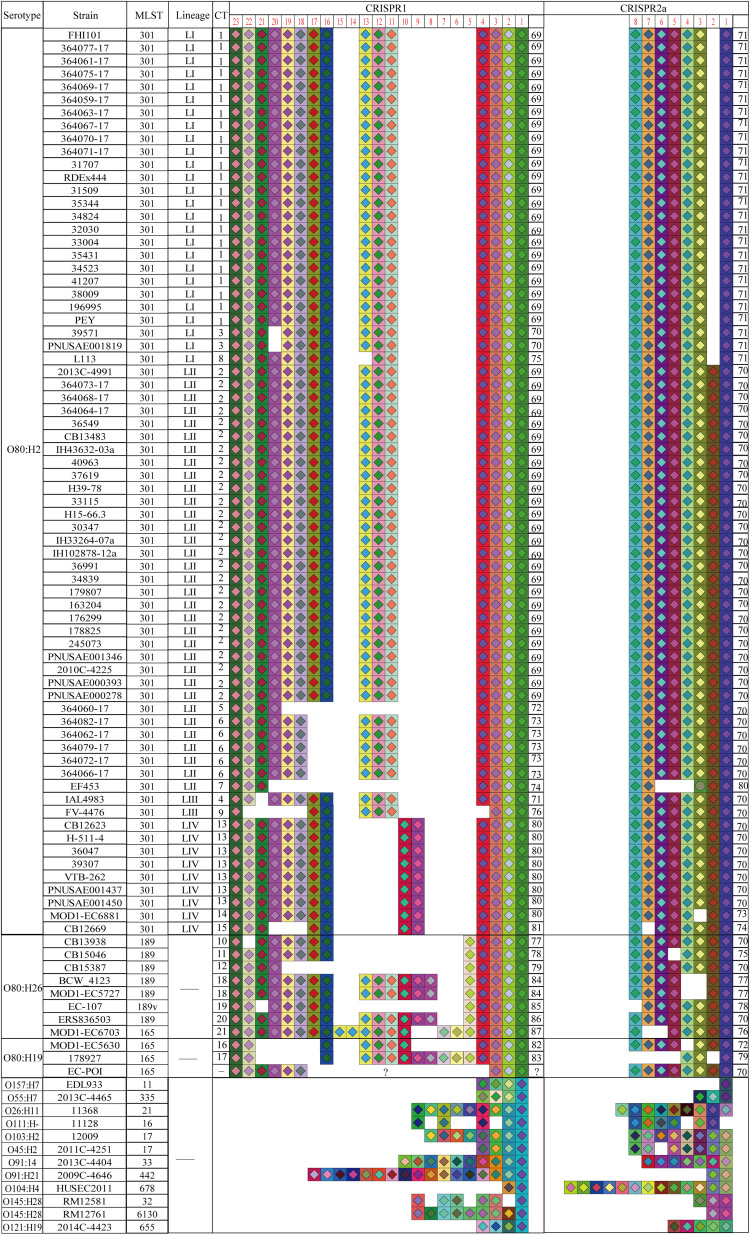
Spacer organizations and arrangements of STEC O80 strains as well as 12 reference STEC strains. Spacer is represented by one square, and each unique spacer sequence is marked as the combination of a unique color and one center symbol. The symbol in the center indicates the length of spacer (diamond for 32 bp and circle for 31 bp). Spacer numbering is initiated at the ancestral end (right) toward the most recently acquired spacers (left) per strain. The left side represents CRISPR1, and the right represents CRISPR2 for the same strain in the same line. The serotype, MLST, CRISPR lineage, CRISPR type, CRISPR1 allele, and CRISPR2 allele are displayed in the corresponding column.

Strain EC-POI was excluded for subsequent CRISPR typing analysis because CRISPR1 array in the strain was truncated due to the troubling of sequencing. Thus, a total of 80 O80 strains were typed based on CRISPR1 and CRISPR2a loci. The spacer arrangements of CRISPR1 and CRISPR2a loci in the 80 O80 strains were summarized in Figure 2. A total of 20 different spacers arranged in 19 alleles were found in CRISPR1 of the 80 O80 strains, which resulted in 23.75% (19/80) alleles diversity (Supplementary Table S2). Of them, the most common allele (CRISPR1 allele 69) was found 49 times (61.25% of the isolates), whereas 14 alleles were present only once. Among the others, two alleles were found twice, one allele was found five times, and one allele was found eight times. Within CRISPR1, the alleles had between 7 and 21 spacers (mean ± standard deviation, 14.44 ± 2.01). All the spacers in CRISPR1 had not been previously described, and therefore, all CRISPR1 alleles were new.

In comparison, CRISPR2a array was more conserved than CRISPR1. CRISPR2a contained 9 different spacers arranged in 11 alleles, which resulted in 13.75% allele diversity in 80 O80 strains analyzed (Supplementary Table S3). CRISPR2a allele 70 was the most prevalent, which was present 44 times, followed by CRISPR2a allele 71 (26 times). The two predominant alleles accounted for 87.5% (70/80) of the isolates. Among the remaining isolates, eight alleles were present once, and one allele was found twice. Each allele in CRISPR2a harbored between 3 and 8 spacers (7.4 ± 0.91), and four spacers were not previously identified. The four newly discovered spacers were all located at the proximal end of leader sequence, and therefore, all the CRISPR2a alleles were also new.

In combination, the CRISPR1 and CRISPR2a alleles formed 21 different CTs that had not been identified in previous investigations. The discriminatory power (discriminatory index) of CT among 80 O80 strains were 0.7966, which means that there should be a 79.66% probability that two unrelated strains can be separated by the CT method. In contrast, the discriminatory powers of serotyping and MLST were 0.2238 and 0.2271, respectively. Hence, the CRISPR typing method displayed greater discriminatory power than serotyping and MLST.

**FIGURE 3:**
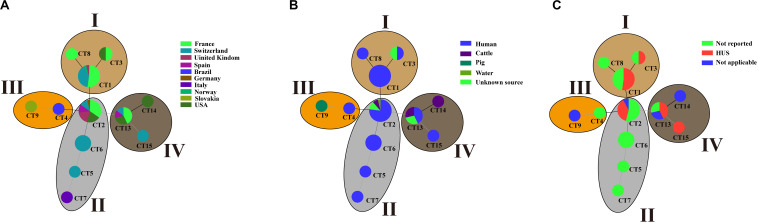
Minimum spanning tree of CTs for 70 STEC O80:H2 strains by geographical region **(A)**, host source **(B)**, or disease status **(C)**. Each nodal point represents one CT, and the size represents the number of strains within the CT, whereas the branch and distribution of all the CTs indicate the relationship among these CTs. Different geographical region **(A)**, host sources **(B)**, or disease status **(C)** correspond to a color indicated in the legend in the right upper side.

A total of 12 CTs were detected in 70 O80:H2 strains (Figure 3). Among all the CTs, CT2 was the most prevalent CT, which accounted for 37.14% (26/70) of the O80:H2 isolates. CT2 was distributed in strains from six countries (France, Switzerland, Germany, Spain, United Kingdom, and United States) and mainly from three sources (human, cattle, and water; Figures 3A,B). The second most prevalent CT was CT1, which was detected in 23 strains (32.86%, 23/70) isolated from human and from four countries (France, Switzerland, Norway, and United Kingdom). In this study, 27 strains were reported to be associated with HUS (Figure 3C). Of them, 22 strains had CT1 (44.44%, 12/27) or CT2 (37.04%, 10/27), which accounted for 81.48% of the reported HUS-related isolates.

Cluster analysis showed that 12 CTs of O80:H2 strains were further grouped into four lineages (LI, LII, LIII, and LIV; Figure 3). The top two predominant CTs, CT1 and CT2, were located in lineage LI and LII, respectively. In all, 33 strains (47.14%, 33/70) formed the largest lineage LII (CT2, CT5, CT6, and CT7), which included 27 human isolates (81.82%, 27/33), 2 cattle isolates, 1 water isolate, and 3 isolates of unknown source. The second largest lineage LI covered three CTs (CT1, CT3, and CT8) with 26 strains, which contained 25 human isolates (96.16%, 25/26) and one isolate of unknown source. Nine strains belonged to the lineage LIV, which included CT13, CT14, and CT15. Among them, four strains were from human, three strains were from cattle, and two strains were of unknown source. Lineage LIII (CT4 and CT9) harbored two strains, which were isolated from human and pig, respectively.

We found that spacer deletion was the main driver of CRISPR divergence when further comparing the spacer composition among strains (Figure 2). For instance, lineage LI differed from LII by a spacer deletion at position 2 in CRISPR2. The spacers at position 10 to 13 in CRISPR1 of O80:H2 strains also had different degree of deletions when compared with four O80:H26 strains and two O80:H19 strains.

### Relationship Among Clustered Regularly Interspaced Short Palindromic Repeat Typing, Multi-Locus Sequence Typing, and Serotyping

To explore the relationship among CRISPR typing, MLST, and serotyping, we examined the organizations and arrangements of spacers between O80 strains and 12 reference STEC strains (Figure 2). The spacer contents and orders in CRISPR2a arrays were highly conserved within all the O80 strains (except strain EF453 O80:H2), irrespective of H-antigen typing. Notably, all the O80 strains shared an ancestral spacer (located at position 1 in CRISPR1), thereby indicating a common ancestor. However, the shared ancestral spacer by O80 strains had a two-nucleotide difference from the 12 reference STEC strains. The difference in ancestral spacer might be important evidence of lineage divergence. Hence, it raised the possibility that O80 strains had undergone a different evolutionary path from other prevalent STEC strains, which was consistent with the cluster by MLST (CC165; Figure 1).

**FIGURE 4:**
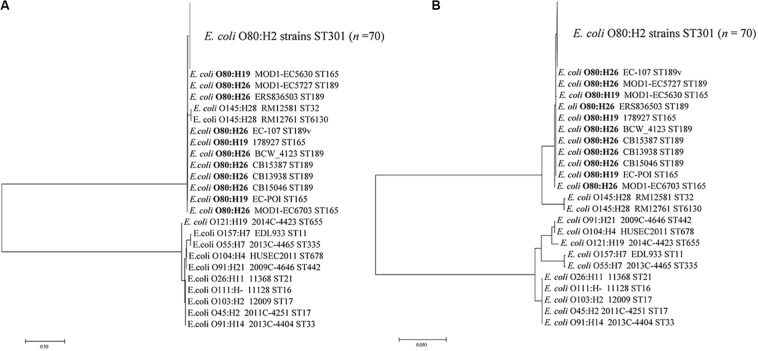
Phylogenetic analysis of STEC O80 strains based on *cas1* gene as well as 12 reference STEC strains. The sequences of *cas1* gene from STEC O80 strains and 12 reference STEC strains are concatenated to construct a neighbor-joining tree **(A)** and maximum likelihood tree **(B)**.

We further analyzed the CRISPR spacer variability among three H antigen types (H2, H19, and H26) and among four MLST types (ST301, ST165, ST189, and ST189v) within O80 strains. As shown in Figure 2, CRISPR typing could divide strains of the same ST or the same serotype into small units. However, there were no shared CTs among the three H antigen types or among four MLST types, which was instrumental for differentiating serotypes or MLST types. Interestingly, all the O80:H26 strains and one O80:H19 strain differed from all the O80:H2 strains by the spacer at position 5 in CRISPR1. We speculated that O80:H26 strains might be phylogenetically closer to O80:H19 than O80:H2. Considering the co-evolve of CRISPR spacer and *cas* gene, a phylogenetic tree based on *cas1* gene was constructed to assess the phylogenetical relationship of the three serotypes. As shown in Figure 4, O80:H26 strains clustered together with O80:H19 strains based on *cas1*.

### Association Among Clustered Regularly Interspaced Short Palindromic Repeat Typing, Whole-Genome Single Nucleotide Polymorphism Typing and Virulence Gene Profiles

**FIGURE 5:**
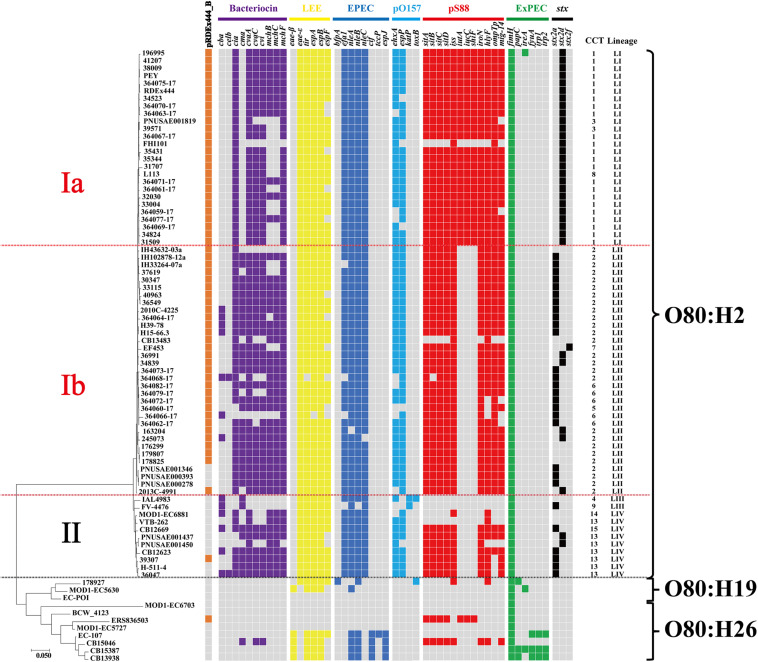
Phylogenetic relationship of all O80 strains based on wgSNPs analysis using CSI Phylogeny 1.4 on the CGE server. Presence of virulence genes or plasmid pRDEx444_B are indicated in cell colors: gray (absence) and brown, purple, yellow, steel blue, light blue, red, green, and black (presence). Genes related to bacteriocin are indicated in purple. Virulence genes related to LEE are indicated in yellow, and virulence genes related to EPEC but not related to LEE are indicated in steel blue. Virulence genes related to pO157 and Shiga toxin are indicated in light blue and black, respectively. Virulence genes related to pS88 are indicated in red, and virulence genes related to ExPEC but not related to pS88 are indicated in green. The CT and CRISPR lineage of each strain are indicated on the right side.

To better understand the phylogenetic relatedness of O80 strains, we extracted the wgSNPs of all O80 strains using the CSI Phylogeny tool with the *E. coli* K-12 MG1655 (accession number: NC_000913.3) as reference (Figure 5). Similar to the earlier mentioned cluster by *cas1* gene, O80:H26 strains were not closely related to O80:H2 but to O80:H19.

As previously reported by Cointe et al. (2018), O80:H2 strains were clustered into three well-supported clusters (Ia, Ib, and II) distinguished by the presence of one cryptic plasmid (plasmid pRDEx444_B) and differentiated virulence gene profiles (Figure 5). Notably, CRISPR typing showed the high accordance with wgSNP typing. Lineage LI and LII of CRISPR typing corresponded to clusters Ia and Ib of wgSNP typing, respectively, and lineages LIII and LIV corresponded to cluster II. Moreover, all strains in lineages LI and LII, except three, harbored the plasmid pRDEx444_B, whereas all strains in lineages LIII and LIV, except one, lacked the plasmid.

We next investigated the relationship between the presence of virulence gene determinants and CTs. As shown in Figure 5, all the strains in lineage LI (except strain FHI101) differed from lineage LII by the presence of three virulence genes (*iutA*, *iucC*, and *shiF*) related to plasmid pS88. Lineage LIII was devoid of all virulent determinants related to plasmid pS88 in comparison with the other three lineages. Additionally, the distribution of three *stx* subtypes (*stx2a*, *stx2d*, and *stx2f*) among four CRISPR lineages was uneven. The positive rate (100%, 26/26) of *stx2d* in lineage LI was significantly higher than that in the other three lineages (lineages LII, LIII, and LIV; *P* < 0.05; Supplementary Figure S1A). However, there was no statistically significant difference between the incidence of HUS in lineage LI (50%, 13/26) and lineage LII (30.30%, 10/33; *P* = 0.124; Supplementary Figure S1B). Overall, there were some relationships between CRISPR lineages and virulence gene profiles.

## Discussion

The structure and function of CRISPR/Cas system have been well studied in multiple species since it was first discovered in *E. coli* in 1987 (Ishino et al., 1987). Before the revelation of its biological and molecular mechanisms, the variable nature of CRISPR has enabled its application in subtyping and detection of strains. At present, several CRISPR-based typing strategies have been established, such as spoligotyping in *M. tuberculosis* (van Soolingen et al., 1998), CRISPR typing, CRISPR locus spacer pair typing, and CRISPR–multi-virulence-locus sequence typing in *Salmonella* spp. (Liu et al., 2011b; Li et al., 2014), and subtyping based on CRISPR locus size in *Yersinia pestis* (Le Fleche et al., 2001). The polymorphism of CRISPR in *E. coli* has also been applied in evolutionary studies and serotype identification. One investigation on 252 STEC strains analyzed the evolutionary divergence of spacers among diverse serotypes and established a CRISPR database to typing STEC strains (Yin et al., 2013). Additionally, CRISPR-based typing has been applied to evaluate genetic diversity of STEC serogroup O91 and O113 strains (Feng et al., 2014, 2017). However, the potential of CRISPR-based genotyping for emerging STEC serogroup O80 has not been evaluated yet.

In this study, a total of 12, 7, and 2 CTs were identified in O80:H2, O80:H26, and O80:H19 strains, respectively. According to the results, CRISPR typing provided discrimination between variants within an ST and showed a better resolution. Likewise, CRISPR typing could divide 65 O113:H21 strains from multiple sources into 50 different CTs (Feng et al., 2014). The highest possible resolution aids in accurately tracking specific strains and correctly identifying infection sources during a single outbreak. In *Salmonella typhimurium* outbreak investigations, the combination of CRISPR typing and other typing methods provided comparable resolution with PFGE and could correctly identify outbreak-related strains (Shariat et al., 2013). Therefore, the better discriminatory power of CRISPR typing may make it more useful than MLST and serotyping in future outbreak surveys related to STEC O80 strains.

Whole-genome sequence typing provides unprecedented discriminatory power, which is favored by more and more epidemiologists in outbreak investigation (Quainoo et al., 2017). Nevertheless, it is one challenging job for most microbiologists and clinicians to analyze WGS data from sequencing platform. Moreover, WGST is still an unaffordable outbreak analysis method for most laboratories in developing countries, although sequencing costs are continuing to fall. Thus, new efforts should be made to develop the alternatives to WGST to perform outbreak investigations in labs without access to WGST. A retrospective investigation in *Salmonella enterica* has illustrated that CRISPR–multi-virulence-locus sequence typing can correctly identify 12 out of 16 outbreak clusters defined by WGST (Deng et al., 2015). Moreover, another investigation found that the clustering based on CRISPR spacer was associated with the lineage constructed by WGST (Li et al., 2018). Similarly, we also found that the clustering by CRISPR typing was related to the group by wgSNP typing. This relationship suggests that CRISPR-based genotyping is superior to other typing methodologies (i.e., MLST and serotyping) in outbreak investigations where WGST is not accessible.

However, one ideal typing technology not only provides excellent discriminatory power required by outbreak analysis but also offers appropriate concordance used for tracing epidemiological changes of certain lineages over a long period of time. Previous investigations have demonstrated an association between CRISPR region and serotyping (Toro et al., 2014). Given that serotyping is still the primary classification scheme for STEC, CRISPR typing may be a complement to serotyping in future STEC surveillance. Indeed, CRISPR typing has been used for a comparative analysis of non-O157 STEC infections between two states in the USA over a 7-year time period, thereby demonstrating the utility of CRISPR typing in long-term epidemiology (Blankenship et al., 2020). In the present study, we found that O80 strains shared a conserved ancestral spacer in CRISPR1 and contained conserved spacer arrangements in CRISPR2. Combined with the cluster results of *cas1* gene and MLST (CC165), it could be concluded that O80 strains constitute a unique clade different from other prevalent STEC serotypes. Previously performed wgSNP typing also demonstrated the high clonality of the O80 strains (Cointe et al., 2018). Additionally, we further confirmed the conservation and specificity of CRISPR spacer composition in STEC O80:H2 strains through a comparative analysis with published investigation (Yin et al., 2013). Thus, it is possible to design a CRISPR-targeting polymerase chain reaction protocol to diagnosis STEC O80:H2 strains and to conduct long-term epidemiological surveillance of this pathogen.

Outbreak-related or pathogenic isolates are more likely to attract attention and are sequenced and submitted to public databases, thus resulting in bias in genomic data. In this study, most of STEC O80:H2 strains were isolated from humans in Europe, especially in France and Switzerland (Figures 3A,B). The dominant CTs, CT1 and CT2, were shared by human isolates from multiple European countries. According to the limited information provided by published papers, almost half of the strains (22/49, 44.90%) within CT1 and CT2 were reported to cause HUS. The incomplete information at least indicated the importance of the two CTs in public health. An investigation in Belgium suggested that contact with cattle might be an important source or transmission route of STEC infections caused by O80:H2 strains (De Rauw et al., 2019). We observed that CT2 and CT13 were shared by human and cattle isolates, which further suggested that there may be the potential transmission of STEC O80:H2 between human and cattle.

The adaptive ability of CRISPR/Cas system led us to speculate that the spacer compositions in CRISPR would identify source-associated clonal populations because of the unique phage pool in each source (Vale and Little, 2010; Liu et al., 2011a, b). Thus, CRISPR typing would be able to trace specific CTs back to their host origin, which has been confirmed in *S. enteritidis* (Liu et al., 2011a, b). Here, we found that some CTs were only detected in human, CT9s were only detected in pig, and CT14s were only detected in cattle. However, it cannot draw a definite conclusion because the dataset in this study was not randomly collected but strongly skewed toward human. Thus, further epidemiological investigations of large samples are required to test the speculation.

Previous study showed that STEC strains with higher pathogenicity contained fewer spacers than those with lower pathogenicity (Toro et al., 2014). In the current study, we also identified a relationship between CRISPR lineages and virulence gene profiles. Compared with lineage LII, lineage LI acquired three virulence genes (*iutA*, *iucC*, and *shiF*) related to plasmid pS88. The three virulence genes are associated with the biosynthesis of aerobactin and involved in the pathogenesis of extraintestinal *E. coli* (Karami et al., 2017). Furthermore, as a predicator for severe clinical outcome of STEC infection (Bielaszewska et al., 2006), *stx2d* gene exhibited a significantly higher positive rate in lineage LI than lineage LII. These findings may hint that strains in lineage LI harbored higher potential pathogenicity than lineage LII. Based on the general evolution mode from non-pathogenic to pathogenic bacteria, it could be hypothesized that lineage LI might have been evolved from lineage LII. Yin et al. (2013) analyzed the stepwise evolution mode from O55:H7 to O157:H7 and found that spacer deletion was the major driver of CRISPR diversity. Similarly, we found that lineage LI lacked the spacer at position 2 in CRISPR2a compared with lineage LII. In an evolutionary sense, the timeline of strains evolution seemed to coincide with the events of spacer deletions. Besides, we observed that O80:H26 and O80:H19 strains were devoid of most virulence genes related to O80:H2 (Figure 5). O80:H2 strains had the deletions of spacers at position 10 to 13 in CRISPR1 when compared with 4 O80:H26 and 2 O80:H19 strains (Figure 2). Based on spacer deletion, it was assumed that O80:H26 and O80:H19 strains might be the ancestor of O80:H2 strains. Cointe et al. (2018) also reported that one O80:H19 strain might represent the ancestral precursor of all the O80 strains. This strain was also included in the current study to characterize CRISPR array. Unfortunately, we cannot further test this hypothesis because its CRISPR array was truncated in this study. Overall, CRISPR typing provides new insights into strains microevolution.

## Conclusion

In conclusion, CRISPR typing is one valuable molecular typing tool that can provide important information for microevolution and evolutionary trajectories of STEC O80 strains. The identification of CT2 and CT13 in human and cattle isolates suggested that they should be regarded as a matter of public health concern and be continuously monitored to prevent the transmission to human.

## Data Availability Statement

All datasets generated for this study are included in the article/Supplementary Material.

## Author Contributions

JL, GD, and SC designed the study. JL and YXu analyzed data and wrote the manuscript. HY, YXi, and LO collected some data. All authors read and approved the final manuscript.

## Conflict of Interest

The authors declare that the research was conducted in the absence of any commercial or financial relationships that could be construed as a potential conflict of interest.

## Footnotes

1 http://enterobase.warwick.ac.uk/

2 https://bigsdb.pasteur.fr/ecoli/

3 http://www.shigatox.net/ecmlst

4 https://www.ncbi.nlm.nih.gov/

5 http://rast.nmpdr.org/

6 http://www.genomicepidemiology.org

7 https://cge.cbs.dtu.dk/services/CSIPhylogeny/

8 http://www.mgc.ac.cn/cgi-bin/VFs/v5/main.cgi?func=VFanalyzer

## Supplementary Material

FIGURE S1 The distribution of *stx2d* gene **(A)** and HUS cases **(B)** among four CRISPR lineages. Significant difference (*p* value < 0.05) are indicated by an asterisk (ns, no significance).
