# Supplementary figures and images for: Utilization of Clustered Regularly Interspaced Short Palindromic Repeats to Genotype Escherichia coli Serogroup O80
Source: Front Microbiol. 2020 Jul 23;11:1708. doi: 10.3389/fmicb.2020.01708 (PMC7390953; doi:10.3389/fmicb.2020.01708)

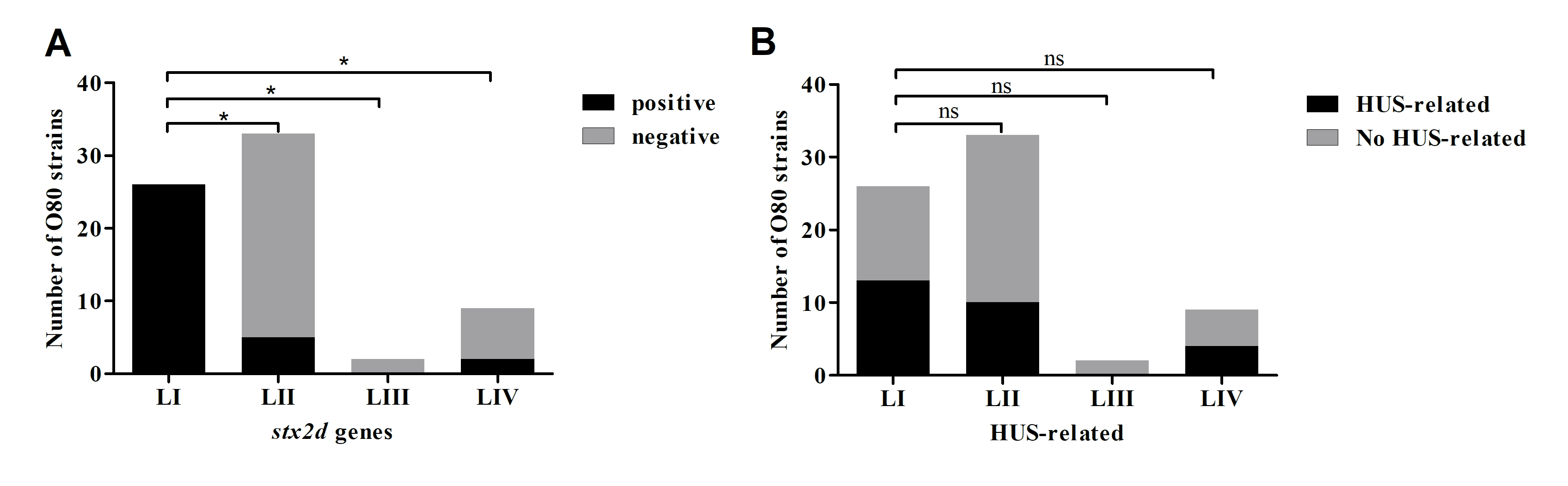

Supplement: FIGURE S1 — The distribution of stx2d gene (A) and HUS cases (B) among four CRISPR lineages. Significant difference (p value < 0.05) are indicated by an asterisk (ns, no significance). [file Image_1.TIF]
